# Limited predatory effects on infaunal macrobenthos community patterns in intertidal soft‐bottom of Arctic coasts

**DOI:** 10.1002/ece3.9779

**Published:** 2023-01-24

**Authors:** María José Díaz, Christian Buschbaum, Paul E. Renaud, Nelson Valdivia, Markus Molis

**Affiliations:** ^1^ Alfred Wegener Institut Helmholtz‐Zentrum für Polar‐ und Meeresforschung Bremerhaven Germany; ^2^ Laboratory of Aquatic Environmental Research (LACER), Centro de Estudios Avanzados, HUB AMBIENTAL UPLA Universidad de Playa Ancha Valparaíso Chile; ^3^ Alfred Wegener Institut, Helmholtz‐Zentrum für Polar‐ und Meeresforschung Wadden Sea Station Sylt List/Sylt Germany; ^4^ Akvaplan‐niva Fram Centre for Climate and the Environment Tromsø Norway; ^5^ University Centre in Svalbard Longyearbyen Norway; ^6^ Centro FONDAP de Investigaciones en Dinámica de Ecosistemas Marinos de Altas Latitudes Santiago Chile; ^7^ Instituto de Ciencias Marinas y Limnológicas, Facultad de Ciencias Universidad Austral de Chile Valdivia Chile; ^8^ UiT The Arctic University of Norway Tromsø Norway

**Keywords:** benthos, biodiversity, consumption, polar region, soft‐bottom habitat, species interactions

## Abstract

Predation shapes marine benthic communities and affects prey species population dynamics in tropic and temperate coastal systems. However, information on its magnitude in systematically understudied Arctic coastal habitats is scarce. To test predation effects on the diversity and structure of Arctic benthic communities, we conducted caging experiments in which consumers were excluded from plots at two intertidal sedimentary sites in Svalbard (Longyearbyen and Thiisbukta) for 2.5 months. Unmanipulated areas served as controls and partial (open) cages were used to estimate potential cage effects. At the end of the experiment, we took one sediment core from each plot and quantified total biomass and the number of each encountered taxon. At both sites, the experimental exclusion of predators slightly changed the species composition of communities and had negligible effects on biomass, total abundance, species richness, evenness, and Shannon Index. In addition, we found evidence for cage effects, and spatial variability in the intensity of the predation effects was identified. Our study suggests that predators have limited effects on the structure of the studied intertidal macrobenthic Arctic communities, which is different from coastal soft‐bottom ecosystems at lower latitudes.

## Graphical abstract

Predation is a key modifier of community dynamics, but information on its magnitude on community regulation in the systematically understudied Arctic coastal habitats. To test the magnitude and direction of the effects of predation on the structure of Arctic benthic communities, we conducted caging experiments in which consumers were excluded from plots at two sites in Svalbard, Arctic. This study suggests that consumers have limited effects on the structure and functioning of the intertidal benthic Arctic communities.

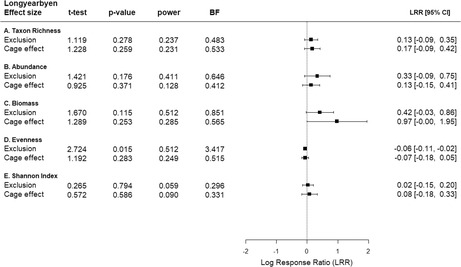

## 1 INTRODUCTION

A key question in ecology is which factors control the diversity and structure of communities. Research on community dynamics is of great interest and has practical scope, for example, for ecosystem conservation and management, preservation of ecosystem services, and the prediction of the response of ecological communities to climate change (Paine et al., 2018; Thompson et al., 2020). Past research showed that both abiotic and biotic factors are important drivers of community structure and function (Wallingford & Sorte, 2019), and knowledge of these drivers is especially needed for polar ecosystems, as climate change is predicted to be strongest at high latitudes (IPCC, 2019).

For coastal Arctic habitats, a number of studies has evaluated the role of abiotic factors in shaping spatial and temporal patterns in taxa distributions, community structure, and taxonomic composition (reviewed in Molis et al., 2019). Ice scouring (Conlan & Kvitek, 2005; Laudien et al., 2007), meltwater discharge (Jerosch et al., 2018), and sedimentation (Veit‐Köhler et al., 2008) have received considerable attention. However, biotic interactions known to affect the dynamics and structuring of temperate soft‐bottom communities, such as bioturbation, facilitation, and consumption (Ambrose Jr, 1984; Wilson, 1990), have been rarely addressed experimentally at higher latitudes. In this context, Poore et al. (2012) showed that herbivore impact assessment experiments are not conducted at latitudes north of 60°N.

Predation can strongly modify population dynamics, distribution, and diversity of prey (Guzman et al., 2019), and its role in shaping intertidal soft‐bottom communities in temperate and tropical regions is well‐documented (Freestone et al., 2011; Reise, 1985). However, information regarding the role of consumers on community structure in the Arctic is scarce and cannot be inferred from experiments that were run in the temperate zone. In one of the few experimental field studies of predator effects on Arctic benthos, Petrowski et al. (2016) showed that the community structure of a subtidal soft‐bottom community in Kongsfjorden (western Svalbard) was less affected by the consumption of epibenthic predators than by bioturbation of the sediment‐reworking lugworm *Arenicola marina*.

The lack of information calls for empirical and experimental studies that have to be conducted in Arctic coastal regions because most knowledge on interactions and population dynamics in benthic Arctic coastal systems is hitherto based on observational studies (reviewed in Molis et al., 2019). However, manipulative field experiments are crucial and necessary to investigate underlying mechanisms of observed community patterns (Molis et al., 2019; Petrowski et al., 2016; Volkenborn & Reise, 2007).

Changes in environmental conditions due to climate warming may alter the strength and direction of biotic interactions (Monaco et al., 2016; Silliman & He, 2018; Wallingford & Sorte, 2019). This may also be the case for predator–prey relationships in Arctic coastal ecosystems (Molis et al., 2019). The current predation pressure from epibenthic predators might change in a warmer Arctic due to an increase in the abundance and activity of resident predators and the northward expansion of predatory fish (Eriksen et al., 2012; Fagerli et al., 2014). For example, Eriksen et al. (2012) show that small arctic fish such as *Myoxocephalus quadricornis* (Linnaeus, 1789), which feeds on small fish, bottom crustaceans, and worms, moved northwards from the area of occupancy in warm years in the Arctic Sea during 29 years (1980–2009). Continued warm periods in the Arctic may promote a changing role for consumers, and ecosystem functioning may be modified. To predict how the ecosystem will react to a warmer Arctic, more information on the current role of consumers in Arctic communities is essential.

Therefore, this study assessed the effects of predation on diversity, community structure, and functional characteristics in Arctic marine soft‐bottom intertidal habitats through manipulative field experiments. In detail, we measured benthic taxa richness, total abundance, and biomass with and without experimental exclusion of predators.

## 2 MATERIALS AND METHODS

### 2.1 Study sites

**FIGURE 1:**
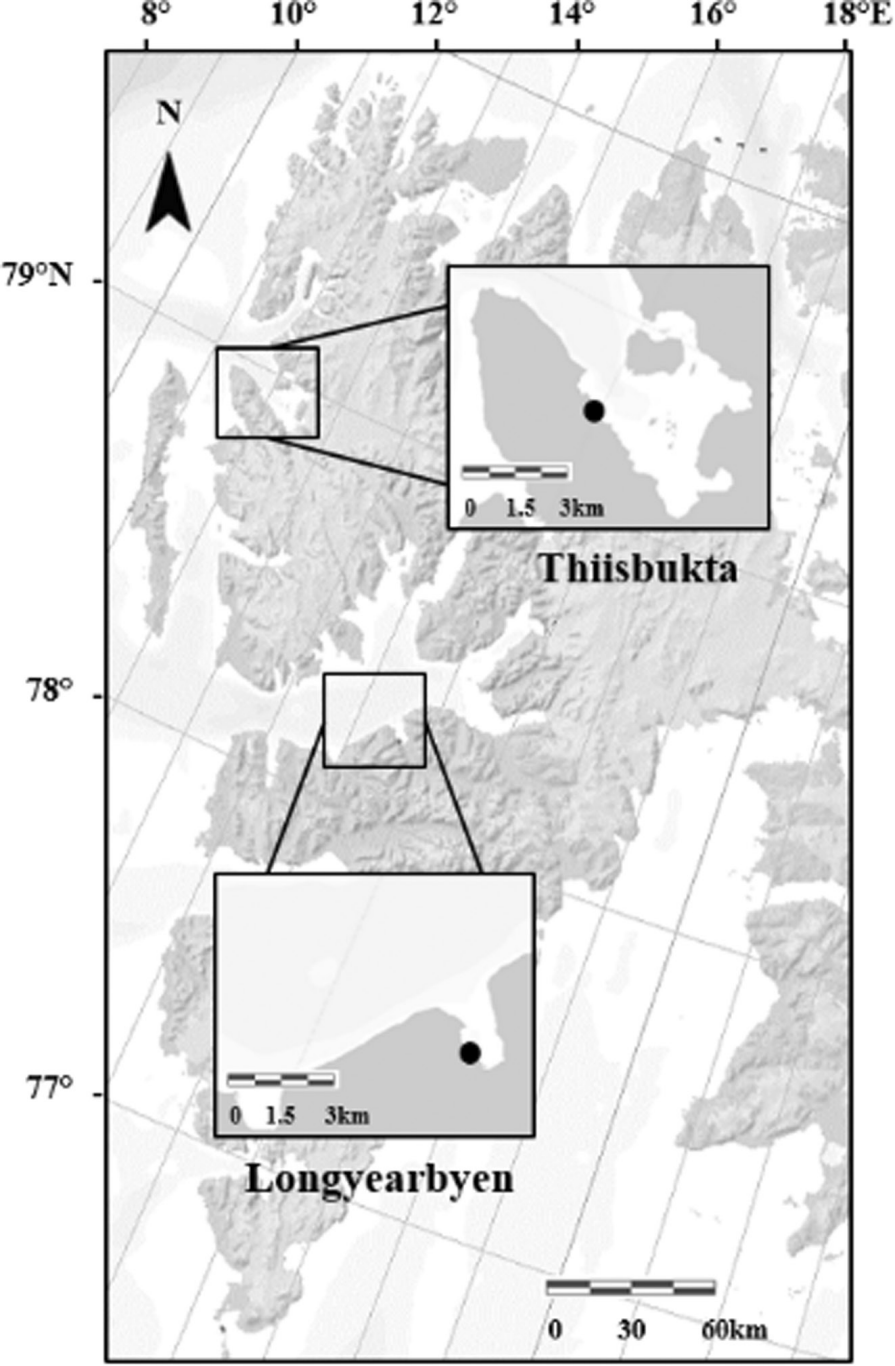
Map of the Svalbard archipelago, with the study sites, Longyearbyen and Thiisbukta, marked with black dots (Norwegian Polar Institute/https://geokart.npolar.no/).

We used two study sites on the west coast of Svalbard for our investigations. One study site was near Longyearbyen located in Adventfjorden (78.21° N, 15.6° E; Figure 1). Adventfjorden is a marine inlet (8.3 km long, 3.4 km wide), which is also influenced by the water bodies of Isfjorden and two rivers (Adventelva and Longyearelva) that cause salinity variations (Zajączkowski, 2008) and an increase in organic matter during summer (Zajączkowski & Włodarska‐Kowalczuk, 2007). Mobile scavenging amphipods, nematodes, and polychaetes belong to the dominating taxonomic groups occurring in the intertidal sedimentary habitat of this fjord (Nygård et al., 2012; Pawłowska et al., 2011), and some of the shorebirds present in the intertidal, for example, *Somateria mollissima*, *Larus marinus*, *Sterna paradisaea*, and *Cepphus grylle*, are shorebirds that prey in the internareal zones of Longyearbyen (Fauchald et al., 2015).

The second study site called Thiisbukta is located in Kongsfjorden, a 30‐km‐long fjord (78.92° N, 11.9° E; Figure 1). Drainage of several rivers into the fjord causes an input of organic material and sediment but also salinity variations from 10 to 33 psu (Svendsen et al., 2002). The intertidal soft‐bottom of Thiisbukta is dominated by oligochaetes, the polychaetes (*Scoloplos armiger* and *Euchone analis*) and bivalves (*Liocyma fluctuosa* and *Macoma* sp.) (McMahon et al., 2006). In terms of potential predators in the study area, common fish species on the soft‐bottoms of the Svalbard coast are *Anisarchus medius* and *Lumpenus lampraeteformis* (Wienerroither et al., 2011), they feed on benthic invertebrates such as amphipods, bottom‐dwelling crustaceans, polychaetes, and larval stages of fish (Eriksen et al., 2012; Wienerroither et al., 2011). Juvenile *Myoxocephalus scorpius* are also considered potential predators on benthic invertebrates on shallow bottoms in Arctic marine waters (Berge & Nahrgang, 2013). Although the information on abundance and composition is scarce, *M. scorpius* was found to be one of the most abundant species (74.9%) in the shallow waters of Kongsfjorden, Svalbard (Brand & Fischer, 2016)

### 2.2 Experimental design, setup, and sampling

**FIGURE 2:**
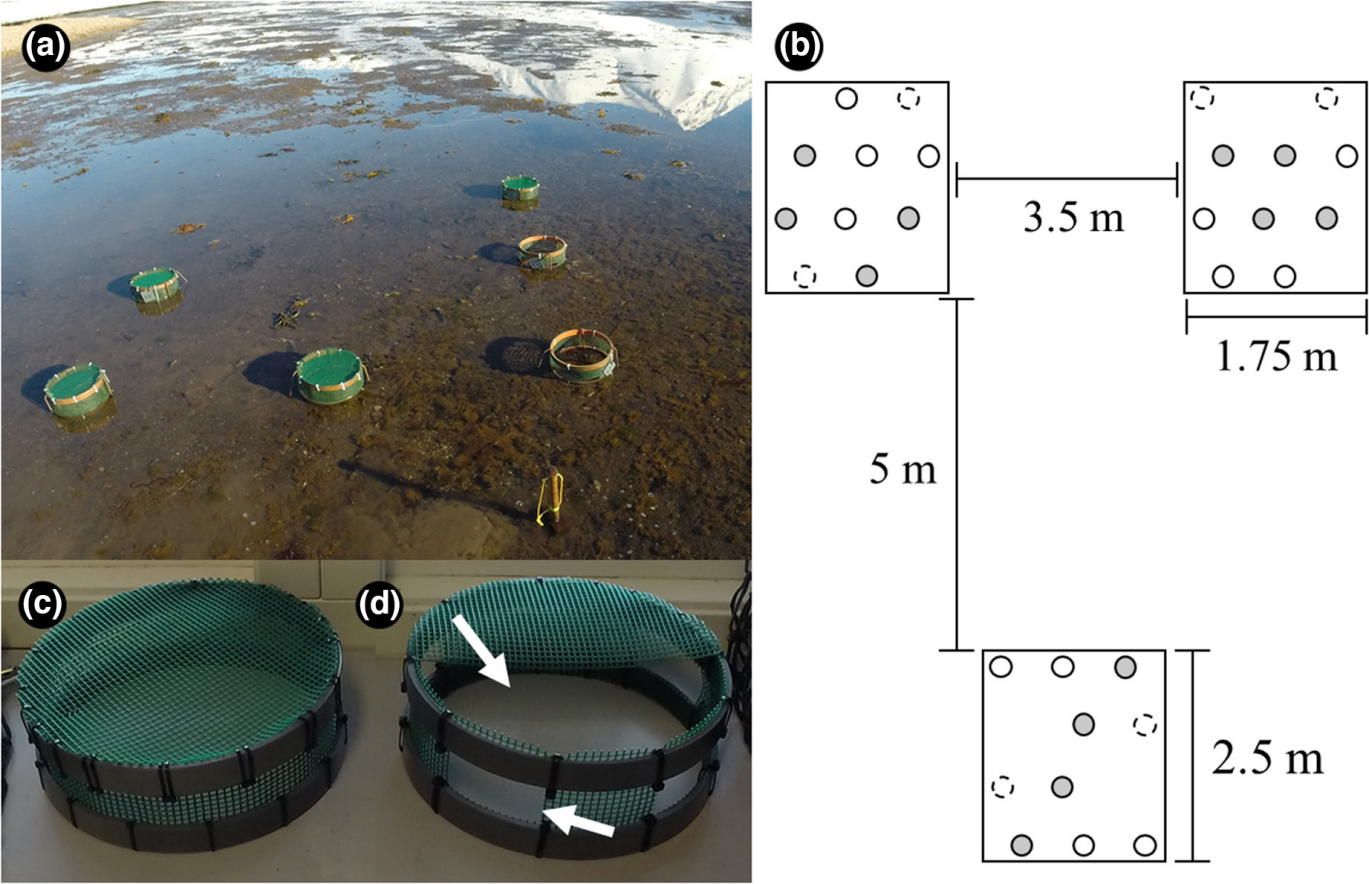
Experimental design and set‐up. (a) Example of one block with randomised allocation of treatments. (b) Dimensions and distribution of the experimental units in the blocks; grey circles (full cage), white circles (unmanipulated area), and dotted circles (partial cage). (c) Full cage to test for “exclusion predator” treatment. (d) Partial cage to test for “cage artefact”, white arrows indicate openings in lid and sidewall.

To investigate the effects of consumption on the infaunal macrobenthic community, identical predator exclusion experiments with randomized block design were conducted at each site. The design included “predator exclusion” as a fixed factor with three treatments: “full cage,” “partial cage,” and “unmanipulated area.” A random factor “block” with three levels was used to quantify whether the effects of predator exclusion varied in space (Figure 2a). The treatments “full cage” and “unmanipulated area” were replicated four times in each block, while the “partial cage” treatment was, due to logistical constraints, replicated twice in each block. This experimental design yielded a total of 30 experimental units (EUs) at each site. Predator exclusion treatments were randomly assigned to 10 EUs per block. Each block covered an area of about 5 m^2^, where EUs were located at a minimum distance of 50 cm (Figure 2b). Each experiment was installed during one low tide at about 1 m above mean low tide level; plots stayed emerged during each low tide for approx. 4 h.

To exclude epibenthic predators (“full cage” treatment), cylindrical cages (25 cm in diameter, 11 cm high) were constructed with a polyethylene mesh (mesh size 0.5 cm), fully covering cage's side and top (Figure 2c). Two PVC rings at the upper and lower end of the cages were used for fixing the mesh. The bottom rings were fully pushed into the sediment (about 5 cm) to limit horizontal movements of organisms, including predatory infauna. To test for cage effects, partial (open) cages were constructed by cutting away half of the mesh at the top and four holes (4 cm × 10 cm) into the cage side to allow consumers to enter and exit the cages (Figure 2d). Each partial and full cage was fixed with three 35 cm iron rods to the seafloor. Unmanipulated, that is, cage‐free, areas served as the control treatment.

Eighty days after the experiment started (May 23, 2017, in Thiisbukta and June 1, 2017, in Longyearbyen), a transparent PVC corer (5.4 cm diameter) was pushed 10 cm deep into the sediment in the center of each EU (= total of 30 samples per site). All samples were kept at 4 °C as intact sediment cores until they were processed in the laboratory of the University Centre in Svalbard (Longyearbyen) or the Marine Laboratory in Ny Ålesund (Thiisbukta) within 4 days after the sampling. Each sample was sieved with a 0.5 mm sieve. All organisms remaining in the sieve were identified to lowest possible taxonomic level using a stereomicroscope, and the number of individuals of each taxon was counted. Pielou's evenness (J), which describes how evenly individuals are distributed across taxa in a sample (Pielou, 1966), was calculated as: J = H′/log S, where H′ is the Shannon index (to natural logarithm) and S is taxon richness (number of species). For each sediment core, the biomass of all organisms per taxon was measured to the nearest 0.001 g with a laboratory balance (Mettler‐Toledo) after drying the organisms in an oven at 60°C to constant weight.

### 2.3 Statistical analyses

We followed the advice of Wasserstein et al. (2019) to report the *p*‐value for all values and considered it as a continuous metric of the probability that the calculated value of a test statistic (or a larger value) occurs by chance, given that the null hypothesis is correct (Crawley, 2013, p. 753). Hence, we neither used the level of α ≤ 0.05 as a dichotomous threshold at which to determine whether a trend is significant nor to label effects as “statistically significant.”

Using the R package “GAD” version 1.1.1 (Sandrini‐Neto & Camargo, 2012), we tested with mixed models ANOVAs whether predation effects (full cages vs. unmanipulated areas) were independent of position within a study site (see ‘E × B’ in Table A1 of the Appendix A for predation effect). Furthermore, we quantified for each univariate response variable the effect size (as log response ratio) of the predation effect using data of fully caged plots and unmanipulated areas, and of the cage effect using data of partially caged plots and unmanipulated areas. We calculated for each univariate response variable five statistical metrics to evaluate the likelihood of an effect. (i) With a Student's *t*‐test, we estimated the value of the test statistic *t* and its probability (*p*), using the function “*t*.test” of the R package “stats” v3.5.1 (Pinheiro et al., 2018). (ii) The power of *t*‐tests was quantified with the “pwr.*t*.test” function of the R package “pwr.2” v1.0 (Lu et al., 2017). (iii) The Bayes factor (BF) as the ratio between the likelihood of data given the alternative hypothesis divided by the likelihood of data given the null hypothesis (Beard et al., 2016). The Bayes factor was calculated with the function “ttest.tstat” from the R “BayesFactor” package v0.9.12–4.2 (Morey & Rouder, 2018). For the interpretation of the Bayes factor, the categories established using the factor ranks determined by Lee and Wagenmakers (2014) were used. (v) The average log response ratio (LRR) was calculated as the decimal logarithm of the quotient of the mean treatment (either fully caged or partially cage) versus the mean control (unmanipulated area), subsequently plotted with its 95% confidence interval (CI) using the “forest” and “scalc” functions of the R package “metafor" v2.4–0 (Viechtbauer, 2019).

Shapiro–Wilks test and quantile–quantile plots were used to check for normality of residuals. Furthermore, Cochran's test and standardized residual‐vs‐fit values were used to test for homogeneity of variances, using the “C.test” function of the R package “GAD” v1.1.1 and graphical exploration of residuals‐vs.‐adjusted‐values plots (Crawley, 2012; Sandrini‐Neto & Camargo, 2012), respectively. The data were fourth root‐transformed when heteroscedasticity of the residuals was registered. Heteroscedasticity increases the type II error rate and therefore should only be taken into account when treatment effects occur (Underwood, 1997).

To test the effects of manipulations on community structure, we analyzed separately for each site relative abundances of macrofauna using Permuted Multivariate Analyses of Variance (PERMANOVA; Anderson, 2001) based on Bray–Curtis dissimilarities. The use of relative abundances provides an unbiased measure on compositional differences by excluding differences in overall counts (Greenacre, 2018). The factors were Treatment (fixed, three levels), Block (random, three levels), and the Treatment × Block interaction. The analyses used 9999 permutations to calculate the *p*‐value for each model term. Permuted Multivariate Analyses of Variances were conducted with the “adonis" function of the R package “vegan” v2. 5–6 (Oksanen et al., 2018). When the *p*‐value of Treatment × Block was >.25, the analysis was repeated after pooling the variance of the interaction term with the residual variance of the full model (Quinn & Keough, 2002). We generated a Principal Components Analysis (PCA) that were plotted with the “plot” function of R “base” package to illustrate (i) treatment effects along the first two principal components explaining most of the variation of the data and (ii) values for the most influential taxa. All analyses were conducted in the R environment, version 3.6.1 (R Core Team, 2019).

## 3 RESULTS

### 3.1 Characterization of the soft‐bottom community

In total, 25 taxa were identified (11 at Longyearbyen and 24 at Thiisbukta). Both sites had several taxa in common, although Thiisbukta reported more individuals in almost all taxa than Longyearbyen. Taxon richness in Thiisbukta was, on average, 52% greater than in Longyearbyen. At both sites, the soft‐bottom fauna was dominated by polychaetes. In total, six (55% of total species number) and 13 (54% of total species number) polychaete taxa were encountered at Longyearbyen and Thiisbukta, respectively (Table 1).

**TABLE 1:** Total abundance (*n*), mean total plot abundance (Mean), proportional abundance (PROP) of each taxon found in the samples (21 cm^2^ area) taken from the fully (full cage) and partially caged (partial cage) plots as well as from the unmanaged (control) areas at the end of the 80‐day experiment in Longyearbyen and Thiisbukta. Empty cells indicate an absence of organisms. *n* = 12.

Phylum/class	Taxon	Longyearbyen									Thiisbukta								
		Control			Full cage			Partial cage			Control			Full cage			Partial cage		
		*n*	Mean	PROP	*n*	Mean	PROP	*n*	Mean	PROP	*n*	Mean	PROP	*n*	Mean	PROP	*n*	Mean	PROP
Nematoda	Nematoda indet.				5	0.42	0.03	2	0.33	0.03	9	0.75	0.01	15	1.25	0.02	4	0.67	0.01
Nemertea	Nemertea indet.	1	0.08	0.01	7	0.58	0.04	1	0.17	0.01	5	0.42	0.003	33	2.75	0.04	7	1.17	0.02
Priapulida	*Priapulus caudatus*										3	0.25	0.003	8	0.67	0.01	5	0.83	0.01
Holothuroidea	*Chiridota laevis*										1	0.08	0.001						
Bivalvia	*Axinopsida orbiculata*										3	0.25	0.003	2	0.17	0.002	2	0.33	0.005
	*Liocyma fluctuosa*										21	1.75	0.02	15	1.25	0.01	7	1.17	0.02
	*Macoma* sp.										6	0.50	0.01	6	0.50	0.01	3	0.50	0.01
Malacostraca	Amphipoda indet.	1	0.08	0.02							5	0.42	0.01	1	0.17	0.002	1	0.17	0.002
	*Caprella linearis*													6	0.50	0.01			
Hexanauplia	Copepoda indet.	1	0.08	0.02	2	0.17	0.01				9	0.75	0.01	28	2.33	0.02	10	1.67	0.02
Clitellata	Oligochaeta indet.	13	1.08	0.08	25	2.08	0.14	7	1.17	0.09	192	16	0.19	84	7	0.07	21	3.50	0.05
Polychaeta	*Bradabyssa villosa*													3	0.5	0.02	3	0.50	0.01
	*Capitella capitata*	38	3.17	0.30	32	2.67	0.18	12	2.00	0.16	74	6.17	0.08	84	7	0.09	29	4.83	0.07
	*Chaetozone setosa*										12	1	0.01	6	0.5	0.01	2	0.33	0.005
	*Euchone analis*										538	44.83	0.49	618	51.5	0.49	259	40.67	0.62
	*Harmothoe imbricate*													1	0.08	0.001			
	*Maldanidae* sp.										12	1	0.01	16	1.33	0.02	2	0.33	0.005
	*Marenzelleria wireni*																		
	*Ophelia rathkei*										2	0.17	0.001	2	0.17	0.001			
	*Pholoe assimilis*													1	0.08	0.001			
	*Phyllodoce groenlandica*				1	0.01	0.01												
	*Polydora* sp.	3	0.25	0.01				2	0.33	0.03	86	7.17	0.09	117	9.75	0.11	30	5.00	0.07
	*Pygospio* cf. *elegans*	43	3.58	0.31	66	5.5	0.34	33	5.50	0.43	2	0.17	0.002	12	1	0.01	7	1.17	0.02
	*Scoloplos armiger*				2	0.17	0.004	2	0.33	0.03	58	4.83	0.06	36	3	0.04	11	1.83	0.03
	*Spio armata*	35	2.92	0.26	48	4	0.25	18	3.00	0.23	7	0.58	0.01	50	4.17	0.05	12	2.00	0.03
	*Travisia forbesii*										5	0.42	0.01	5	0.42	0.01			

*Note*: Thiisbukta. Empty cells indicate an absence of organisms. *n* = 12.

### 3.2 Predator effects

Longyearbyen: Four taxa, *Pygospio* sp., *Capitella capitata*, *Spio armata*, and oligochaetes, accounted for more than 90% of the total abundance. The exclusion of predators increased the abundance of oligochaetes, *Pygospio* sp., and *S. armata* on average by 200, 54, and 37%, respectively, compared with unmanipulated areas. By contrast, partially caged plots in the same taxa resulted in an average decrease of 50, 23, and 51%, respectively, compared with unmanaged areas. The abundance of *C. capitata* decreased strongly in partially caged areas compared with unmanaged areas (Table 1).

**FIGURE 3:**
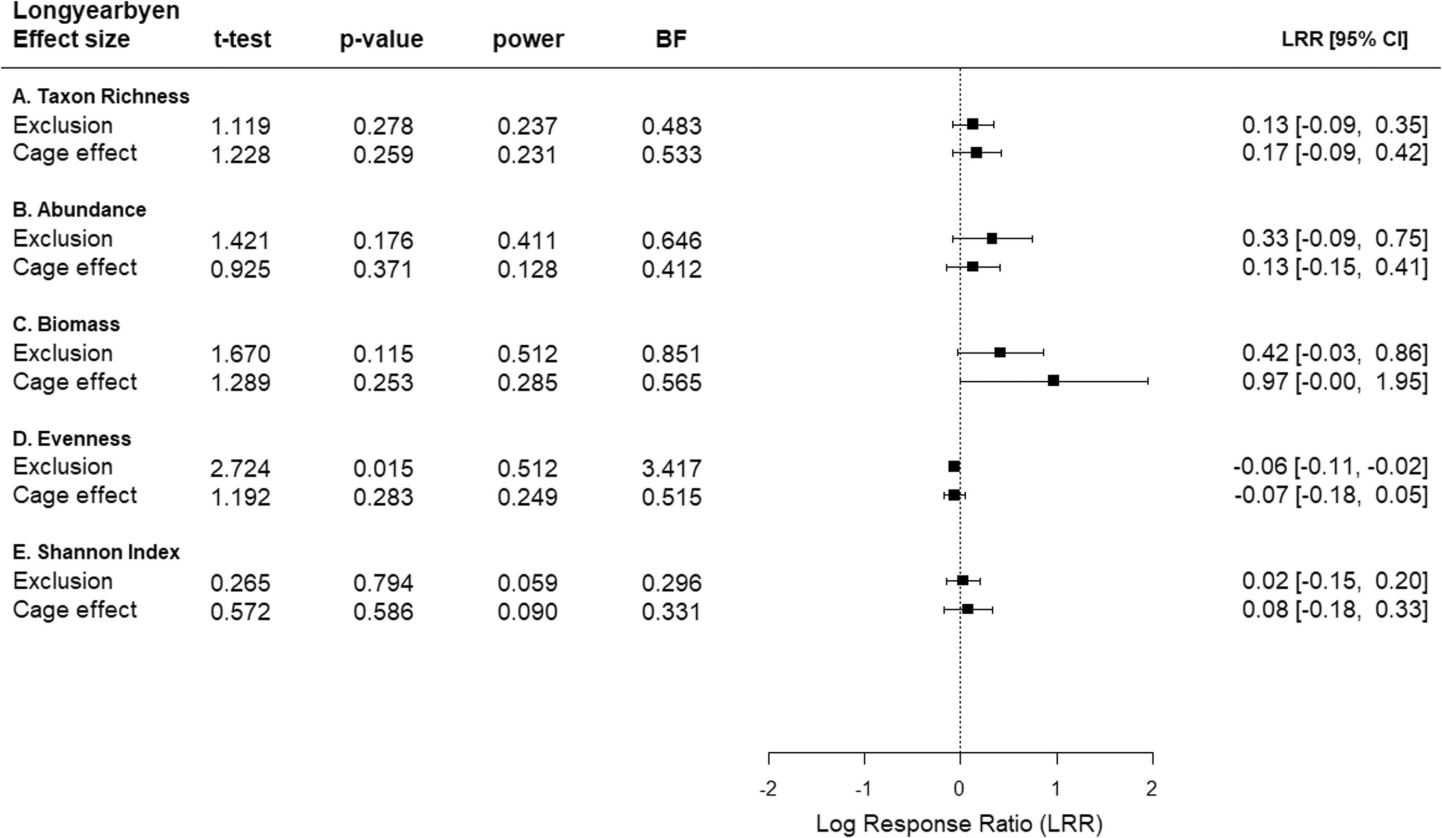
Longyearbyen. Summary of statistical analyses of univariate responses. *t*‐test = statistic of Student's *t*‐test, *p*‐value = probability of test statistic *t*, power = probability of making a type II error (Student's *t*‐test), BF = Bayes factor as evidence for the alternative hypothesis. Mean (square) and 95 % confidence interval (horizontal whiskers) of log effect ratios (LRR) for quantifying the effect of (i) predator exclusion (full cage vs unmanipulated area), (ii) cage (partial cage vs unmanipulated area), for five (A–E) responses. Dashed line = level of no effect, *n* = 12.

The high probability of the *F*‐statistic for the “Exclusion × Block” interaction of all response variables measured in Longyearbyen suggests that the main effects of predator exclusion were unlikely to depend on the location of plots (Table A1). The effects of the predator exclusion treatment were negligible because the magnitude of the exclusion effect was similar to that we found in open cages for most response variables (Figure 3). Predator exclusion negatively affected plot evenness to a slight magnitude (Figure 3). This effect was supported by a low probability of the *t*‐statistic (*p* = .015), a high test power = 0.512, and the Bayes factor suggested that evenness data occurred 3.417 times more likely in a model that includes predator exclusion (Figure 3; Table 2).

**TABLE 2:** Longyearbyen. Summary of statistical analyses of univariate responses.

Response	Effect	Shap	Coch
Taxon Richness	Exclusion	0.012	0.086
	Cage effect	0.104	0.591
Abundance	Exclusion (T)	0.005	0.009
	Cage effect	0.270	0.221
Biomass	Exclusion (T)	0.025	0.018
	Cage effect (T)	<0.001	<0.001
Evenness	Exclusion (T)	0.027	0.018
	Cage effect (T)	<0.001	0.007
Shannon Index	Exclusion	0.129	0.235
	Cage effect	0.467	0.440

*Note*: *n* = 12.

Abbreviations: (T), square root transformed data; Coch, *p*‐value of Cochran's test; Shap, *p*‐value of Shapiro–Wilks test for normality.

Thiisbukta: Seven taxa, that is, *Euchone analis*, oligochaetes, *Polydora* sp., *C. capitata*, *Scoloplos armiger*, *Liocyma fluctuosa*, and copepods comprised >80% of the total abundance. Predator exclusion resulted in an increase in abundance of *C. capitata, E. analis*, and *Polydora* sp. by an average, 13, 15, and 36%, respectively, relative to unmanipulated areas. Contrarily, the abundance of these taxa decreased in partially caged plots by, on average, 61, 52, and 65%, respectively, compared with unmanipulated areas. Moreover, the abundance of *L. fluctuosa, S. armiger*, and oligochaetes was, on average, 29, 38, and 56%, respectively, lower in areas where predators were excluded than in unmanipulated areas. Likewise, the abundances of these taxa decreased by 67, 81, and 89%, respectively, in the partially caged plots compared with the unmanipulated areas. Copepod abundance increased in fully and partially caged areas compared with unmanipulated areas (Table 1).

**FIGURE 4:**
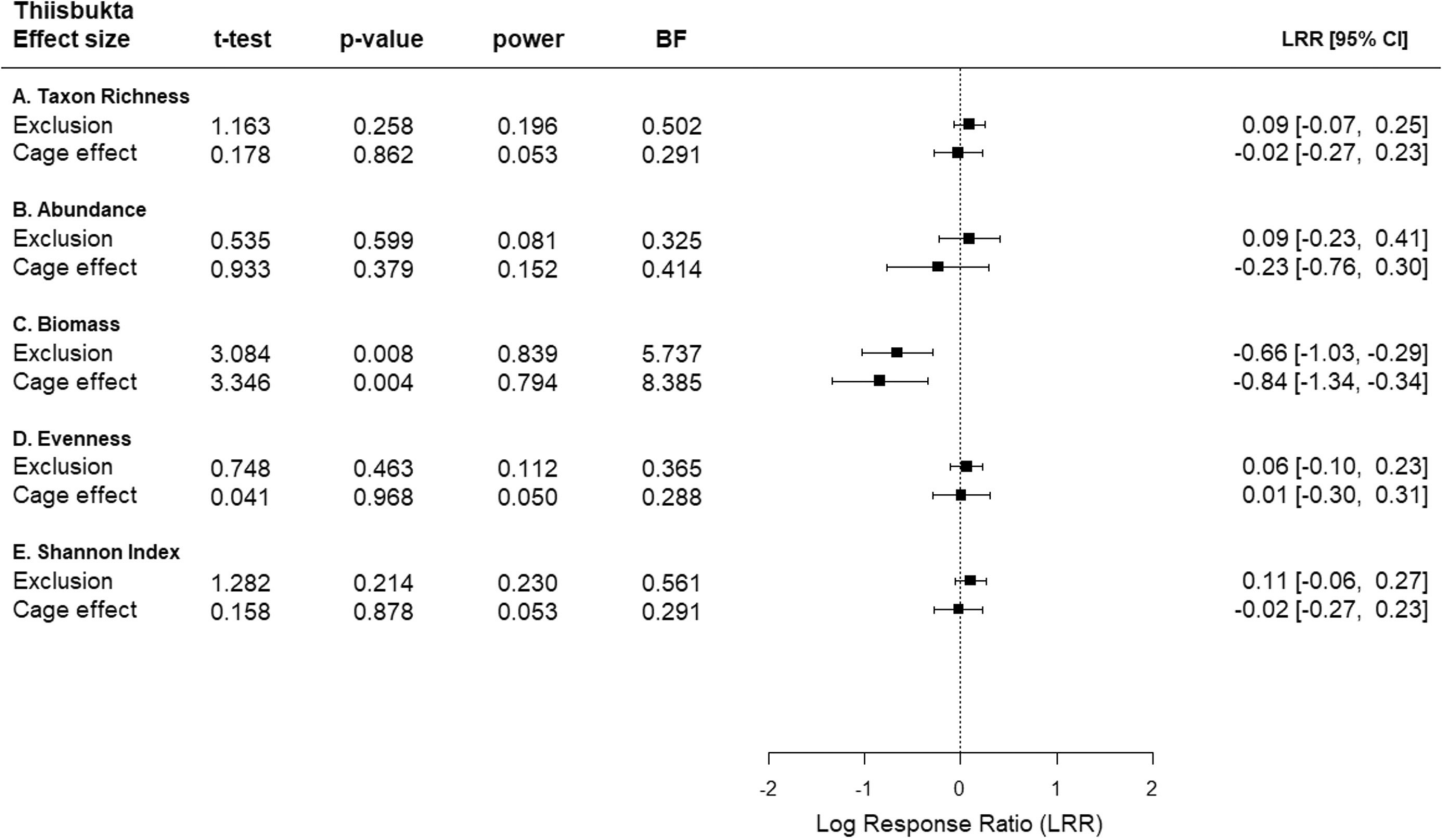
Thiisbukta. Summary of statistical analyses of univariate responses. *t*‐test = statistic of Student's *t*‐test, *p* value = probability of test statistic *t*, power = probability of making a type II error (Student's *t*‐test), BF = Bayes factor as evidence for the alternative hypothesis. Mean (square) and 95 % confidence interval (horizontal whiskers) of log effect ratios (LRR) for quantifying the effect of (i) predator exclusion (full cage vs unmanipulated area), (ii) cage (partial cage vs unmanipulated area), for five (A–E) responses. Dashed line = level of no effect, *n* = 12.

In Thiisbukta, the low probabilities of the *F*‐statistic of the “Exclusion × Block” interaction for both evenness and Shannon index suggest that the effects of predator exclusion on these two response variables depend on the location of plots within the study area (Table A1). The effect sizes of predator exclusion and the cage effect on taxon richness, abundance, evenness, and Shannon index were minor (Figure 4). In Figure 4, it can be seen that the variables mentioned above show similar trends between plots with exclusion treatment, cage effect, and unmanipulated plots. Statistical analyses for these four response variables concerning predation effects showed nonrelevant results, the probability was >20% for the chance‐only *t*‐statistic if the null hypothesis was true (“p” in Figure 4) and a low test power (“power” in Figure 4). Only in the case of biomass was a considerable negative predator exclusion effect observed (LRR = 0.66); this was supported by a low probability of the *t*‐statistic, together with a test power of 85% (Figure 4). In addition, the Bayes factor indicated that the data were 5.7 times more likely under the alternative hypothesis than the null hypothesis (Figure 4). As for the effect of the cage on biomass, the trend was in the same direction and even slightly more substantial than the effect of predator exclusion (Figure 4; Table 3).

**TABLE 3:** Thiisbukta. Summary of statistical analyses of univariate responses.

Response	Effect	Shap	Coch
Taxon richness	Exclusion	0.130	0.434
	Cage effect	0.376	0.593
Abundance	Exclusion	0.151	0.361
	Cage effect	0.978	0.081
Biomass	Exclusion (T)	0.005	0.011
	Cage effect	0.215	0.819
Evenness	Exclusion	0.761	0.356
	Cage effect	0.964	0.391
Shannon index	Exclusion	0.313	0.641
	Cage effect	0.065	0.935

*Note*: *n* = 12.

Abbreviations: (T), square root transformed data; Coch, *p*‐value of Cochran's test; Shap, *p*‐value of Shapiro–Wilks test for normality.

### 3.3 Predator exclusion effects on community structure

**FIGURE 5:**
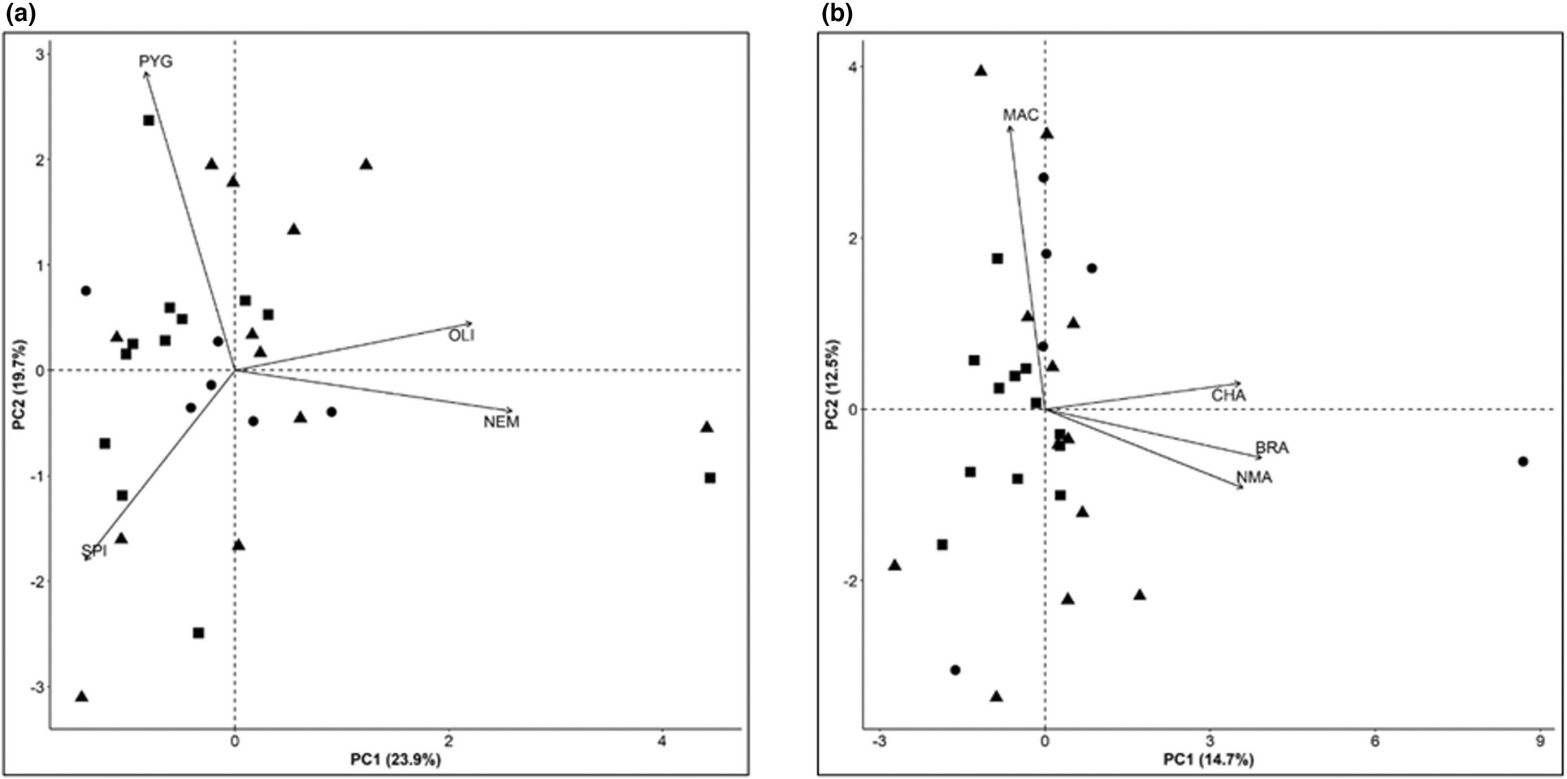
Principal Components Analysis (PCA) showing two principal components explaining in (a) Longyearbyen 43.6% and in (b) Thiisbukta 27.2 % of the total variation in Bray‐Curtis similarity of relative taxon abundances among communities sampled in unmanipulated areas (squares) to partial cages (circles) to full cages (triangles). Loading vectors (black arrows) indicate the four taxa contributing strongest. BRA, Bradabyssa villosa; CHA, Chaetozone setosa; MAC, Macoma sp.; NEM, Nemertea; NMA, Nematodes; OLI, Oligochaeta; PYG, Pygospio sp.; SPI, Spio armata.

The low probability of the *F*‐statistic for the Exclusion × Block interaction term suggests that effects of predator exclusion on species composition depended on the location within the study site where manipulations were applied, for both, Longyearbyen and Thiisbukta (Table 4). In Longyearbyen, the main predation effect was accounted for by the increase in abundance of *Pygospio* sp., oligochaetes, nematodes, and *S. armata* between unmanipulated areas and fully caged plots (Table 1 and Figure 5A). In Thiisbukta, the increase in abundance of *Macoma* sp., *C. setosa*, Nemertea, and *B. villosa* accounted for most of the predator‐removal effect on species composition (Table 1 and Figure 5B).

**TABLE 4:** Summary of PERMANOVA results based on 9999 permutations of Bray–Curtis similarities calculated of relative abundances of taxa.

	Longyearbyen					Thiisbukta			
Source of variance	Df	MS	*F*	*p*	MS_den_	MS	*F*	*p*	MS_den_
Exclusion (E)	1	0.09	1.22	.315	E × B	0.19	3.03	.017	E × B
Block (B)	2	0.10	1.36	.222	E × B	0.06	1.03	.409	E × B
Exclusion × Block	2	0.16	2.15	.029	Resid	0.25	3.98	<.001	Resid
Residual	18	0.08				0.06			

*Note*: Mixed model two‐way analyses (predator exclusion) were reanalyzed if the respective treatment × block interaction showed *p* ≥ .25, by pooling residual variance and that of the interaction term of the full model. MS_den_ indicates MS of the source of variance used as denominator to calculate the *F*‐value. *n* = 12.

Abbreviation: Resid, Residuals.

## 4 DISCUSSION

In this study, predator exclusion resulted in weak effects on all tested univariate response variables. This indicates that predation has only a limited regulatory impact on the studied Artic intertidal soft‐bottom communities. In Thiisbukta, the biomass response was similar in direction and magnitude between the predator exclusion treatment and the cage effect, suggesting that the cage itself and not predation was the cause. Predator exclusion slightly affected the multivariate community structure at both sites; however, this effect was block‐dependent.

In our study, the results of the biomass variable show the effect of the cage on the intertidal benthic community, underestimating the exclusive effect of predation on the infaunal macrobenthos in soft‐bottom communities. Ecologists have used cages for decades in manipulative experiments evaluating predation effects. In assessing the structural effects of cages in intertidal environments, Miller and Gaylord (2007) found a drastic decrease in water flow velocity within cages compared with the velocity of the surrounding water. Due to reduced water flow, the sedimentation rate may increase within the cage, affecting settlement, feeding, or other elements of species performance, thus leading to impacts on benthic community structure (Como et al., 2006; Reise, 1985; Schmidt & Warner, 1984; Smale & Barnes, 2008). Another possible impediment to detecting the effect of predation on the benthic community is the size of the cages. The cages were 25 cm in diameter, which may be insufficient to see an effect on the macrobenthic community, particularly for mobile organisms such as crustaceans and snails that live and move on the surface. In addition, the sampling core (5.4 cm diameter) may be sufficient to determine the effect of predation on the sessile infauna and meiofauna community. Furthermore, a reduced diameter may be sufficient to determine the impact of predators on the macrobenthic community in a sample. However, the results obtained in this research correctly determine the impacts of predation on the minor infaunal and sessile macrobenthic community, excluding the larger and mobile infaunal macrobenthic organisms (e.g., *Onisimus littoralis*, *Gammarus setosus*, *Orchomenella minuta*, and Harpacticoida).

Theoretical models predict that the effects of predation and other biotic interactions are highly dependent on prevailing levels of environmental stress. Thus, predator activity is expected to decrease when subjected to high environmental stress, such as harsh abiotic conditions (Menge & Sutherland, 1987; Scrosati et al., 2011). In intertidal polar coastal regions, the prevalence of ice cover, the abrasive action of icebergs/drift‐ice, and factors such as extreme diurnal and seasonal changes in temperature, light and salinity are considered hostile to most marine taxa (Barnes & Conlan, 2007; Gutt, 2001; Hansen & Haugen, 1989; Wȩsɫawski et al., 1997). This supports the contention that polar intertidal zones are among the most physically disturbed marine environments in the world (Bick & Arlt, 2013; Wȩsɫawski et al., 1997) and organisms living in this area have to deal with these conditions.

Under such abiotic stress, predation may not be expected to structure marine communities at high latitudes (Schemske et al., 2009) and predation is generally concluded to play a minor role in structuring Arctic soft‐bottom communities (Molis et al., 2019; Petrowski et al., 2016; Quijon & Snelgrove, 2005), although few studies have actually been performed. Our research also indicates a low impact of predation on community regulation at two Svalbard intertidal soft‐substrate sites. Similarly, manipulative studies conducted in the White Sea subtidal reveal that predation plays a minor role in structuring the benthic community (e.g., Petrowski et al., 2016; Yakovis & Artemieva, 2015).

Ocean warming and decreasing ice coverage in the Arctic are predicted to result in range expansion (spatial and depth) of resident and immigrant taxa, which may have important direct and indirect implications for interactions among taxa (Josefson & Mokievsky, 2013; Renaud et al., 2015). For example, sea ice serves as habitat and modulates access and life histories of both predators and prey. Its loss can, thus, impact broad elements of the food web via its effects on trophic interactions (Aronson et al., 2007; Renaud et al., 2015; Schachtl, 2013). In the Arctic, warming is expected that boreal congeners of resident intertidal/subtidal predators, hermit crabs (*Pagurus* sp.) and spider crabs (*Hyas* sp.), will expand northward and be recorded more frequently in the Svalbard Archipelago (Balazy et al., 2015; Berge et al., 2009). Increased density and diversity of crustacean predators could lead to a higher predation pressure on the benthic community. This was demonstrated by Bender (2014) in a manipulative study at a subtidal site in the Svalbard Archipelago, in which densities of the crustacean *Hyas araneus* were experimentally increased by a factor of three in comparison with natural crab densities. At higher crab densities, species richness and density of soft‐bottom fauna decreased. Additionally, the community structure was modified.

Our experiments suggest a small spatially variable effect of predator exclusion on taxonomic composition. In particular, taxa such as Nemertea indet., nematodes, and *S. armata* increased in abundance, while polychaetes such as *E. analis* and *C. setosa* decreased in density in predator exclusion plots relative to controls, indicating that some species benefited from predator exclusion while others suffered from this manipulation. This could explain why multivariate, but not univariate, community response variables were affected by predator exclusion. Our results were consistent between sites (no effect on univariate, block × treatment interaction on species composition). Therefore, this is an indication that predation effects at intertidal sites on the west coast of Svalbard appear to be weak for the soft‐bottom microbenthic infaunal community. As global warming continues apace in the Arctic, further field research on biotic interactions is needed to assess the functional consequences of possible range shifts in high‐latitude consumer and prey species.

## AUTHOR CONTRIBUTIONS

**Paul E. Renaud:** Conceptualization (supporting); methodology (supporting); writing – review and editing (supporting). **Nelson Valdivia:** Conceptualization (supporting); visualization (supporting); writing – review and editing (supporting). **María José Díaz:** Formal analysis (equal); methodology (equal); software (equal); visualization (equal); writing – original draft (equal). **Markus Molis:** Conceptualization (equal); data curation (equal); formal analysis (supporting); methodology (equal); supervision (equal); visualization (equal); writing – review and editing (supporting). **Christian Buschbaum:** Conceptualization (equal); data curation (equal); methodology (equal); supervision (equal); writing – review and editing (equal).

## Data Availability

The raw data supporting the conclusions of this article are available on PANGEA https://doi.pangaea.de/10.1594/PANGAEA.943807 upon request.

## APPENDIX A

**TABLE A1:** Longyearbyen and Thiisbukta. Summary of ANOVA results on separate and interactive effects of predator exclusion (fixed) and blocks (random) at the end of the experiment.

Response variable	Source	Longyearbyen				Thiisbukta			
		E	B	E × B	Res	E	B	E × B	Res
Taxon richness	*df*	1	2	2	18	1	2	2	18
	MS	1.50	0.17	1.50	1.28	7.04	7.13	1.30	5.43
	*F*	1.17	0.13	1.17		1.30	1.31	0.24	
	*p*	.293	.879	.332		.270	.294	.791	
Abundance	*df*	1	2	2	18	1	2	2	18
	MS	117	52.17	24.67	62.29	392	7473	457	795
	*F*	1.88	0.84	0.40		0.493	9.41	0.58	
	*p*	.187	.449	.679		.491	.002	.573	
Biomass	df	1	2	2	18	1	2	2	18
	MS	2.7 e‐5	5.4 e‐6	9.1 e‐6	1.0 e‐5	0.14	0.02	0.01	0.02
	*F*	2.65	0.54	0.91		9.40	1.42	0.45	
	*p*	.121	.594	.421		.007	.267	.648	
Evenness	*df*	1	2	2	18	1	2	2	18
	MS	0.02	0.01	0.01	0.00	0.01	0.11	0.03	0.01
	*F*	9.96	2.91	2.86		1.41	14.20	4.47	
	*p*	.006	.081	.084		.251	.000	.027	
Shannon index	*df*	1	2	2	18	1	2	2	18
	MS	0.01	0.04	0.12	0.07	0.16	0.34	0.26	0.06
	*F*	0.07	0.63	1.74		3.00	6.32	4.74	
	*p*	.791	.545	.204		.101	.008	.022	

*Note*: *n* = 12.

Abbreviations: B, block; df, degrees of freedom; E, exclusion; MS, mean square; Res, residual.
